# Insights into Structural Transformation during Oxidative Stabilization of Pan Nanofibers Reinforced with Carbon Nanotubes and Graphene Nanoplatelets

**DOI:** 10.1021/acspolymersau.6c00010

**Published:** 2026-03-26

**Authors:** Felipe Carlos dos Reis, Juliano Marini, Erick Gabriel Ribeiro dos Anjos, Mirabel Cerqueira Rezende, Fabio Roberto Passador

**Affiliations:** † Department of Science and Technology, Federal University of São Paulo (UNIFESP), 330 Talim St., São José dos Campos, São Paulo 12231-280, Brazil; ‡ Graduate Program in Materials Science and Engineering, Federal University of São Carlos (UFSCar), Rodovia Washington Luís, Km 235, São Carlos, São Paulo 13565-905, Brazil

**Keywords:** electrospinning, polyacrylonitrile, oxidative stabilization, carbon nanotubes, graphene nanoplatelets, dielectric properties

## Abstract

To elucidate the influence of oxidative stabilization on structural evolution and functional performance, electrospun PAN-based nanofiber mats reinforced with CNTs (1–2 wt %), GNPs (5–10 wt %), and CNT/GNP hybrids containing 1–2 wt % CNTs combined with 3–9 wt % GNPs were systematically investigated. After stabilization, FT-IR and DSC analyses confirmed enhanced cyclization in the presence of nanofillers, reflected by the increase in conjugation index from 0.76 (neat PAN) to 0.90 for CNT/GNP (2/3 wt %) fibers. The DSC results confirm this through the disappearance of the exothermic peak typically associated with the cyclization reaction. Thermogravimetric results showed a significant rise in char yield, reaching up to 70.2% for the 10 wt % GNP mat at 700 °C. Electromagnetic shielding performance was strongly influenced by the porous architecture of the electrospun mats, resulting in predominantly absorptive attenuation behavior. Nanoparticle incorporation, fiber orientation, and thermal treatment directly influenced the dielectric response, leading to higher thickness-averaged specific shielding effectiveness (TASSE_T_). The best performance was achieved for stabilized sPAN/C2 in the orientation parallel to the incident electromagnetic field (0°), reaching ε_r_ = 18.90, TASSE_T_ = 407.37 dB·cm^2^·g^–1^. Under perpendicular alignment (90°), stabilized sPAN/C2/G8 exhibited ε_r_ = 8.64 and reached TASSE_T_ = 198.48 dB·cm^2^·g^–1^, outperforming single-filler systems in this configuration.

## Graphical abstract

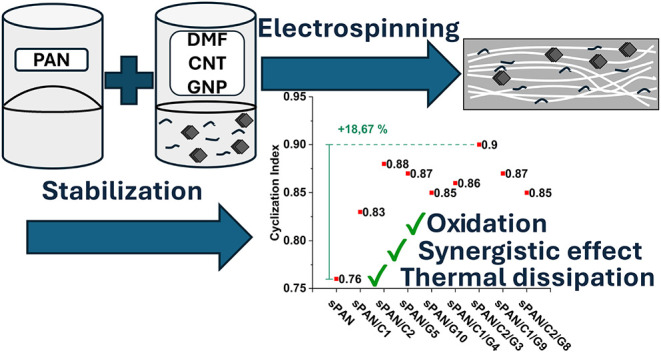

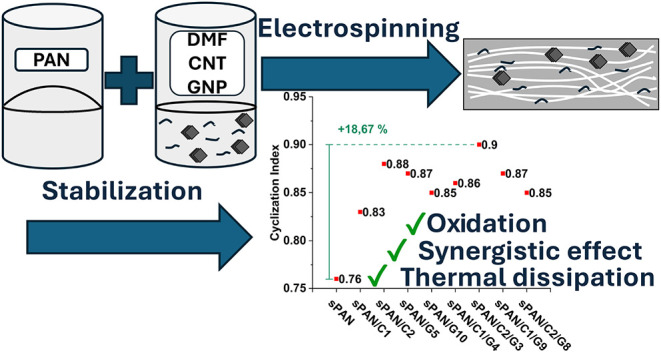

## 1 Introduction

Polyacrylonitrile (PAN) fibers are widely used in everyday applications, including blankets, outdoor awnings, and sweaters.^1^ Beyond these conventional uses, PAN, as a homopolymer or copolymer, is the primary precursor for the production of high-performance carbon fibers.^2^ To tailor the internal morphology and mechanical properties of PAN-based fibers before carbonization, several spinning techniques have been developed, such as dry spinning, melt spinning, wet spinning, and dry-jet wet spinning.^3–5^ Among these methods, wet spinning is the most employed process, due to its versatility in tailoring the structural, mechanical, and thermal properties of fibers.

In recent years, electrospinning has emerged as an attractive alternative, as it allows the fabrication of continuous ultrafine PAN fibers by applying a high electric field between a polymer-solution-filled needle and a grounded collector.^6^ Under the applied field, electrostatic forces overcome surface tension at the needle tip, ejecting a charged liquid jet that undergoes rapid stretching, solvent evaporation, and deposition as micro or nanoscale filaments. This process enables the production of PAN nanofibers with diameters in the order of hundreds of nanometers, offering significant potential advantages over conventional micron-sized precursors.^7^ The substantial reduction in fiber diameter may reduce structural defects and promote a more uniform sheath-core morphology, both of which are key to improving the tensile strength and modulus of the resulting carbon fibers.^8^ Furthermore, the high specific surface area of electrospun nanofibers facilitates heat and mass transfer during stabilization and carbonization, improving reaction efficiency and conversion yield.

These advantages have driven extensive research on electrospun PAN-based carbon nanofibers (CNFs).^9,10^ However, it has been consistently reported that CNFs derived from electrospun precursors exhibit a predominantly turbostratic structure, characterized by higher porosity and a disordered carbon lattice.^11^ This behavior is commonly attributed to relaxation effects caused by residual solvent trapped within the nanofibers, which limits molecular packing during stabilization and carbonization.^12,13^ In addition, the mechanical stresses imposed during electrospinning are nonuniform and substantially less effective at aligning polymer chains than the postspinning drawing process used industrially, resulting in reduced macromolecular orientation.^14^ Consequently, considerable efforts have been devoted to improving both the microstructure and mechanical performance of electrospun CNFs.^15^

One effective strategy involves the incorporation of carbon nanofillers such as carbon nanotubes (CNTs) and graphene nanoplatelets (GNPs) into PAN nanofibers.^16–20^ GNPs consist of stacked graphene layers that form extended two-dimensional platelets, which enhance charge transport and provide numerous sites for polymer–filler interactions.^21^ However, graphene-based fillers typically require higher loadings to achieve significant property enhancements when compared to CNTs. This difference is commonly attributed to the exceptional aspect ratio of CNTs, which enables the formation of percolated networks at low concentrations, facilitating efficient stress transfer and high axial electrical conductivity.^22–24^

The combination of GNPs and CNTs as hybrid nanofiller allows the complementary advantages of both materials to be exploited, often resulting in synergistic improvements in structural and functional properties.^18^ Previous studies have reported that CNT/GNP hybrid systems in PAN nanofibers can reduce the activation energy required for the oxidation and cross-linking during stabilization, improve the orientation of the developing ladder structure, and increase the maximum tensile load sustained during thermal stabilization.^25^ Additionally, the presence of both nanofillers enhances thermal stability and promotes higher char yields, which is advantageous for carbon-rich or thermally resistant materials. Some reports further suggest that carbon nanofillers may initiate partial cyclization during electrospinning, leading to early molecular conversion of PAN and a more efficient structural evolution during subsequent thermal treatments.^26^ At high temperatures, PAN chains tend to organize around carbon nanoparticles, which act as nucleation sites for graphitic domains, thereby promoting increased graphitization and improved mechanical and electrical performance.^17^

Given these pronounced structural and chemical effects, a detailed investigation of the transformations occurring during thermal treatment is essential. Among these processes, oxidative stabilization is particularly important because it results in an infusible structure capable of withstanding high temperatures, required for carbonization. Inadequate stabilization conditions can result in excessive weight loss during carbonization, which inevitably affects the structure and performance of the resulting carbon fibers.^27,28^

During stabilization, PAN fibers are heated in air above the glass transition temperature, inducing shrinkage or stretching due to chain disorientation and concurrent chemical reactions, including cyclization, dehydrogenation, and oxidation. These reactions, together, generate a thermally stable ladder-like structure that serves as a precursor for the turbostratic graphitic domains formed during carbonization.^29^ Stabilized PAN fibers also exhibit intrinsic properties, such as flame resistance and thermal insulation, being commercially used in fire-blocking fabrics for transportation seating, protective clothing for firefighters, and as asbestos substitutes in automotive friction materials.^30–32^

During cyclization, the most important stabilization step,^33^ nitrile groups undergo a first-order exothermic reaction with adjacent units to form an infusible ladder macromolecule.^34^ This transformation is accompanied by dehydrogenation, with the release of water, and the formation of conjugated carbon double bonds, subsequently facilitating molecular rearrangement.^35^ Oxidation constitutes the final stabilization step, in which oxygen radicals attack the cyclized structure. Oxygen incorporation is essential for subsequent carbonization, as it contributes to the development of carbon fiber architecture. Since PAN does not have intrinsic oxygen-containing groups, oxygen needs to diffuse from the surrounding air into the fibers, resulting in oxidation that begins at the fiber surface and propagates toward the core, frequently leading to chemical heterogeneity in the fiber cross-section.^36–38^

Optimum stabilization of PAN fiber requires careful control of processing parameters. Fibers must be adequately annealed to prevent rupture while achieving sufficient elongation, and the initial molecular structure should favor efficient cyclization. For example, PAN homopolymers with high isotacticity generally exhibit faster development of conjugated structures. In contrast, the incorporation of reinforcing fillers can generate competitive effects, as polymer–filler interactions may hinder the proximity of neighboring nitrile groups and slow cyclization kinetics. Therefore, stabilization temperature and time must be carefully optimized to maximize cyclization while minimizing degradation reactions.^39^

In this work, these transformations are investigated using a combination of complementary characterization techniques. The evolution of chemical structure during stabilization is examined by monitoring the reduction of nitrile groups and the formation of new conjugated and C=N functionalities. Thermal behavior is evaluated through the identification of exothermic events associated with cyclization, as well as weight variations linked to dehydrogenation and solvent release. Morphological observations are performed to assess the fiber integrity, surface texture, and porosity development. Furthermore, because changes in chemical bonding and pore structure can significantly affect electromagnetic properties, the dielectric response in the X-band frequency range is also investigated together with impedance spectroscopy. The nanofiber mats studied were prepared using different concentrations of CNTs, GNPs, and CNT/GNP hybrid fillers, with the total nanofiller content maintained below 10 wt % to ensure spinnability and homogeneous dispersion.

## 2 Experimental Section

### 2.1 Materials

Polyacrylonitrile (PAN) was supplied in powder form by Radici Group (Brazil), PAN exhibits a number-average molar mass (Mn) of 267,000 g·mol^–1^ and a density of 1.14 g.cm^–3^.

Graphene nanoplatelets (GNPs) were provided by Cheap Tubes (USA). The GNPs consist of fewer than four graphene layers, with an average thickness below 4 nm, lateral dimensions ranging from 1 to 2 μm, and a purity greater than 99%.

Multiwalled carbon nanotubes (MWCNTs) were supplied by Nanocyl S.A. (Belgium) under the NC7000 grade. These nanotubes exhibit a minimum purity of 90%, an average diameter of 9.5 nm, and an average length of approximately 1.5 μm.

### 2.2 Methods

#### 2.2.1 Electrospinning Process

Neat PAN nanofiber mats were prepared from a 10 wt % PAN solution in *N*,*N*-dimethylformamide (DMF). Flow rate was maintained at 1.5 mL/h. A constant voltage of 13 kV was applied to the system, and the distance between the needle tip and the collector was set at 7 cm. The collector consisted of a rotating metallic drum covered with aluminum foil, operating at a constant speed of 1000 rpm (Figure 1).

**Figure 1:**
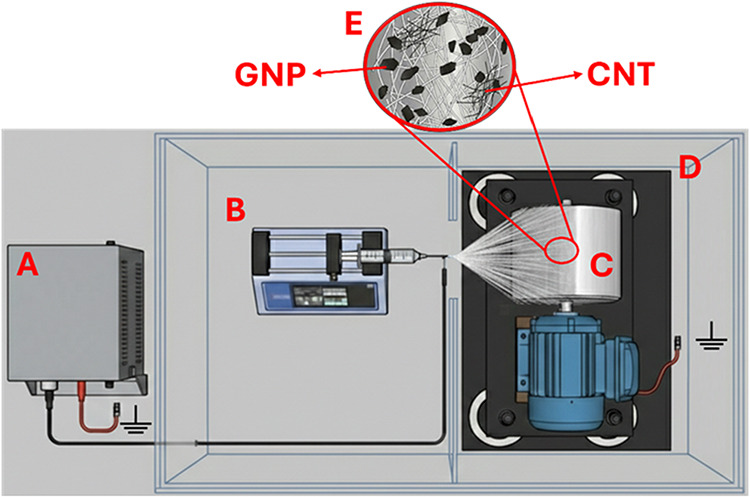
Schematic representation of the electrospinning setup, digitally adapted from experimental photographs using Gemini-assisted rendering, highlighting: (A)—High voltage source; (B)—syringe pump; (C) - collector with aluminum foil; (D)—Acrylic box; (E)—Resultant PAN mat.

For the nanocomposite mats, carbon nanomaterials were first dispersed in DMF by ultrasonication and subsequently mixed with PAN before electrospinning. All nanocomposite solutions were electrospun under the same conditions used for the neat PAN samples. The solid-phase compositions of the electrospun mats are summarized in Table 1, with the total nanofiller content limited to a maximum of 10 wt % to ensure homogeneous dispersion within the polymer matrix.

**Table 1:** Nomenclature and Solid Phase Compositions for Each Sample

sample	PAN (wt %)	CNTs (wt %)	GNPs (wt %)
PAN	100		
PAN/C1	99	1	
PAN/C2	98	2	
PAN/G5	95		5
PAN/G10	90		10
PAN/C1/G4	95	1	4
PAN/C2/G3	95	2	3
PAN/C1/G9	90	1	9
PAN/C2/G8	90	2	8

#### 2.2.2 Oxidative Stabilization

Stabilization was carried out in an FT-HI20–10 P electric furnace (EDG—Brazil) equipped with a cylindrical quartz chamber (Figure 2A). During thermal treatment, the nanofiber mats were mounted on a graphite holder and mechanically constrained by connecting the upper and lower sections of the holder to maintain tensile stress along the fiber direction (Figure 2B). This configuration was employed to promote polymer chain alignment along the fiber axis, thereby enhancing structural integrity during stabilization.

**Figure 2:**
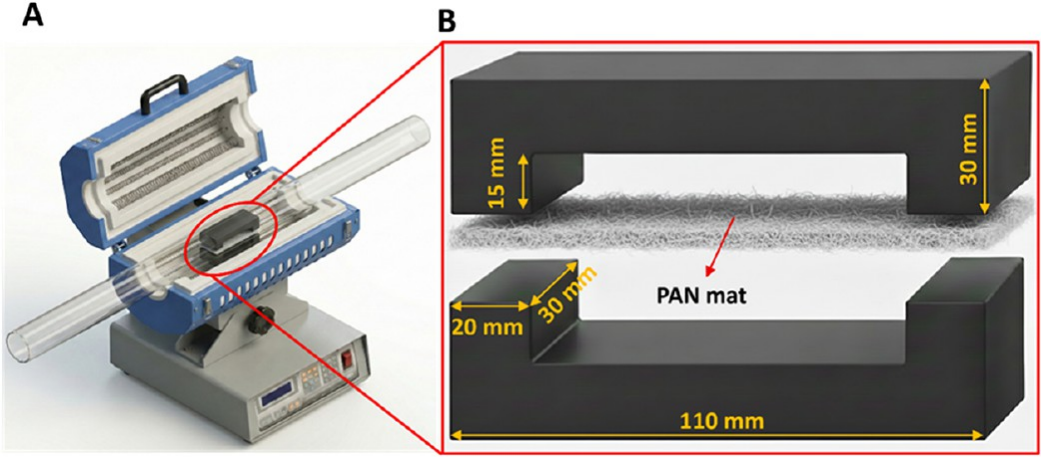
Schematic representation of the thermal treatment equipment, digitally adapted from experimental photographs using Gemini-assisted rendering, highlighting: (A)—Electric furnace; (B)—Graphite holder and its dimensions.

The assembly was positioned at the center of the furnace and heated from room temperature up to 300 °C, corresponding to the furnace set point temperature, at a heating rate of 1 °C min^–1^. Upon reaching this temperature, the samples were held isothermally for 2 h under an oxidizing atmosphere (ambient air). After completion of the thermal treatment, the samples were cooled to room temperature in air. From this point onward, the stabilized samples are identified by the prefix “s”. The stabilization temperature was defined based on preliminary DSC experiments on PAN mats previously heat-treated in an oven at different temperatures, starting at 250 °C, with increments of 25 °C up to 350 °C. Based on the DSC results, the stabilization temperature was selected considering the lowest temperature at which the exothermic peak associated with the cyclization reaction was no longer observed.

### 2.3 Characterization

#### 2.3.1 Carbon Particles Characterization

The carbon-based nanomaterials were characterized by field-emission gun scanning electron microscopy (FEG-SEM), Raman spectroscopy, Fourier transform infrared (FT-IR) spectroscopy, and X-ray diffraction (XRD).

XRD scans were conducted using a Rigaku Ultima IV diffractometer over a 2θ range of 10° to 60°, at a scanning rate of 10°/min. Using Bragg’s law (eq 1) and Scherrer eqs (eq 2), the interplanar distance (*d*_002_) and average thickness size (D_002_) were found, respectively.^40^ The average number of layers was also calculated.

1 $$n\lambda =2d_{002}\sin\theta$$

2 $$D_{002}=\frac{0.9\lambda }{B_{002}\cos\theta }$$

where *n* represents an integer number (*n* = 1, 2, 3), λ is the wavelength of the X-rays (λ = 1.54056 Å), θ is the diffraction angle, *B*_002_ is the full width at half-maximum (FWHM) of the prominent diffraction peak of the graphitic planar structure.

The structural integrity of the carbon materials. Measurements were conducted using a LabRAM HR Evolution spectrometer (HORIBA Scientific), equipped with a helium–neon (He–Ne) laser operating at a wavelength of 532 nm. The spectral region analyzed ranged from 400 to 3000 cm^–1^. From the acquired spectra, the average distance between structural defects (*L*_a_) was estimated using eq 3.^21^

3 $$L_{a}=\left(2,\;4\times 10^{-10}\right)\lambda^{4}{\left(\frac{I_{D}}{I_{G}}\right)}^{-1}$$

where *I*_D_/*I*_G_ is the e ratio between the intensities of the D and G bands.

#### 2.3.2 Field Emission Gun Scanning Electron Microscopy (FEG-SEM)

Morphological characterization of the PAN mats was performed using a TESCAN MIRA3 scanning electron microscope operating at an accelerating voltage of 5 kV. Electrospun mats were cut into small rectangular specimens, detached from the aluminum foil collector, and mounted onto the stubs using double-sided carbon tape.

All samples were sputter-coated with a thin layer of gold to ensure sufficient electrical conductivity for imaging. Fiber diameter distributions were determined using the open-source ImageJ software. For each sample, at least 60 individual fiber diameters were measured from multiple FEG-SEM images to ensure statistical reliability. Measurements were performed by drawing lines perpendicular to the fiber axis.

#### 2.3.3 Thermal Characterization: DSC and TGA

The thermal behavior of the PAN mats were analyzed by differential scanning calorimeter (DSC), using a TA Instruments Q2000 calorimeter under a nitrogen atmosphere, with a flow rate of 50 mL.min^–1^. The thermal program consisted of an initial cooling step from room temperature to −10 °C at a rate of 10 °C.min^–1^, followed by heating from −10 °C up to 350 °C at the same heating rate.

Thermal degradation behavior was investigated by thermogravimetric analysis (TGA) using a TA Instruments Q600 SDT simultaneous thermal analyzer. Approximately 5.0 mg of each sample were placed in alumina crucibles and heated from 25 to 700 °C at a heating rate of 10 °C.min^–1^, under a nitrogen atmosphere with a flow rate of 100 mL.min^–1^.

#### 2.3.4 FT-IR

The surface chemical composition of the mats was investigated by Fourier transform infrared (FT-IR) spectroscopy using the universal attenuated total reflection (UATR) mode. This technique was employed to identify chemical bonds and functional groups within the fiber structure. Spectra were acquired over the range of 4000 to 400 cm^–1^, with a spectral resolution of 4 cm^–1^ and a total of 32 scans, using a PerkinElmer Spectrum One spectrometer.

The intensity of the nitrile absorption band was used to evaluate the degree of thermo-oxidative stabilization through the cyclization index. The cyclization index of the fibers can be calculated according to eq 4.^41^

4 $${\mathrm{CI}}_{FT-IR}=\frac{I_{o}}{I_{n}+I_{o}}$$

where, CI_FT–IR_ represents the cyclization (aromatization) index determined from infrared spectroscopy. *I*_o_ corresponds to the intensity of the absorption band at 1600 cm^–1^, attributed to C=C, and *I*_n_ corresponds to the intensity of the nitrile band (C≡N) at 2240 cm^–1^ measured from the nonstabilized mat.

#### 2.3.5 Electromagnetic Characterization

Measurements of the electrical permittivity (*ε*_r_) of the samples were performed over the frequency range of 8.2 to 12.4 GHz (X-band) and calculated according to eqs 5 and 6. Where the real part of the permittivity (ε′) represents the material’s ability to store electric energy through polarization mechanisms, while the imaginary part (ε″) quantifies the dielectric losses, reflecting the energy dissipated as heat.

5 $$\varepsilon_{r}=\varepsilon^{\prime }-{i\varepsilon }^{\prime \prime }$$

6 $$\left|\varepsilon_{r}\right|=\sqrt{{\left(\varepsilon^{\prime }\right)}^{2}+{\left(\varepsilon^{\prime \prime }\right)}^{2}}$$

The scattering parameters (*S*_11_ and *S*_21_) were experimentally obtained and used to determine the total electromagnetic interference shielding effectiveness (SE_T_). The shielding effectiveness was calculated according to eqs 7–9.^42^ Where SE_R_ is the reflection contribution, and SE_A_ is the absorption contribution.

7 $$SE_{T}=SE_{R}+SE_{A}$$

8 $$SE_{R}=-10\log_{10}\left(1-|S_{11}{|}^{2}\right)$$

9 $$SE_{A}=-10\log_{10}\left(\frac{|S_{21}{|}^{2}}{1-|S_{11}{|}^{2}}\right)$$

To account for the lightweight nature of the electrospun mats, the thickness-averaged specific shielding effectiveness (TASSE) was calculated by normalizing the total shielding effectiveness, and its components by the areal density (grammage) (ρ_A_ given as g·cm^–2^) of the samples, as shown in eq 10 ^43^

10 $$\mathrm{TA}\mathrm{SSE}=\frac{\mathrm{SE}}{\rho_{A}}$$

#### 2.3.6 Electrical Impedance Spectroscopy

Impedance module measurements were obtained by electrical impedance spectroscopy (Solartron SI 1260 Impedance/Gain-phase Analyzer) and applying a 0.5 V voltage in the frequency range of 1 to 10^6^ Hz. These analyses were conducted from the formation of electrodes through the metallization of the specimens with a thin layer of gold/palladium alloy on the two surfaces perpendicular to the direction in which the impedance is performed.^21^

## 3 Results and Discussion

### 3.1 Carbon Nanoparticles Characterization

Carbon nanoparticles can be comprehensively characterized to provide insights into their morphology, surface chemistry, and structural ordering. SEM images (Figures 3A, and 4A) reveal distinct morphologies, where CNTs appear as entangled tubular structures, while GNPs exhibit stacked thin platelet-like sheets due to the layered nature of graphene. FT-IR spectra (Figures 3B, and 4B) show weak absorption bands associated with graphitic domains and surface functional groups, indicating the presence of oxygen-containing species on the carbon surface originating from synthesis or environmental exposure.^44^

**Figure 3:**
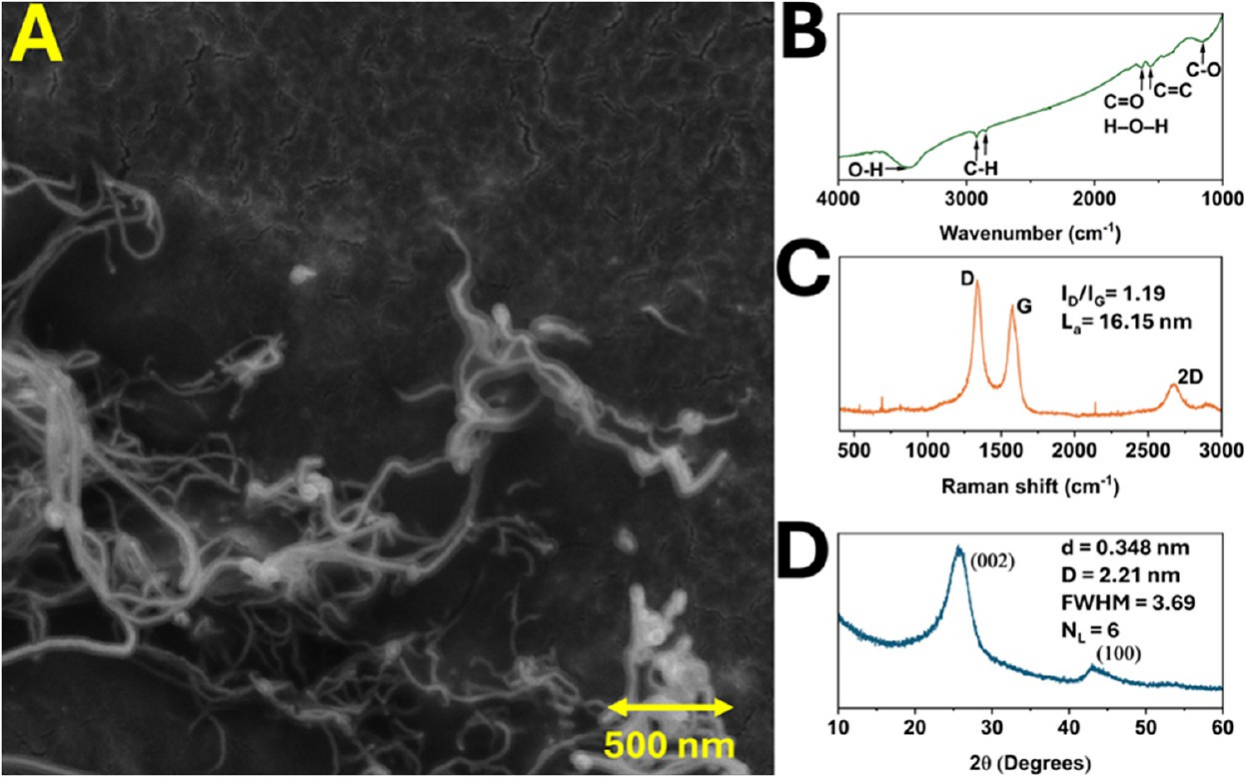
Characterization of CNTs: (A)—SEM image; (B)—FT-IR spectrum; (C)—Raman spectrum; and (D)—XRD pattern.

**Figure 4:**
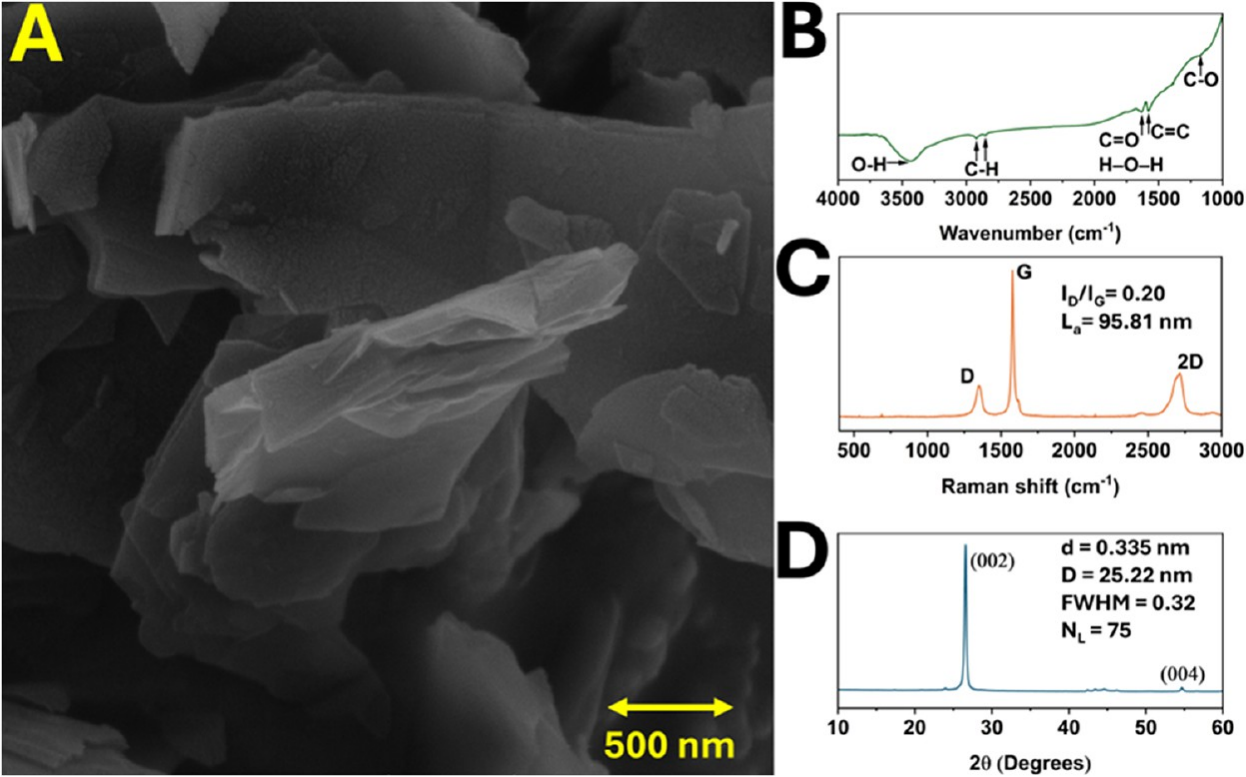
Characterization of GNPs: (A)—SEM image; (B)—FT-IR spectrum; (C)—Raman spectrum; and (D)—XRD pattern.

Raman spectroscopy (Figures 3C, and 4C) shows two major bands, the D band, related to defects in the sp^2^ carbon lattice, and the G band, corresponding to the in-plane vibration of graphitic carbon atoms.^45^ XRD patterns (Figures 3D and 4D) show a distinct diffraction peak corresponding to the (002) plane of graphitic carbon.^46^ From the Raman and XRD analyses, it is also possible to determine important structural parameters such as the average number of graphene layers (*N*_L_), the average interdefect distance (*L*_a_), and the intensity ratio of bands D and G (*I*_D_/*I*_G_). The calculated parameters for CNTs and GNPs are summarized in Figures 3 and 4, respectively, where the characteristic FT-IR vibrational modes are also identified.

### 3.2 Morphological Characterization

Before stabilization, the electrospun PAN mats exhibit a characteristic white coloration and consist of fibers with diameters on the order of hundreds of nanometers, characterized by a largely uniform, smooth, and continuous morphology. The incorporation of CNTs and GNPs significantly modifies the neat PAN solution properties, most notably viscosity and electrical conductivity, which directly influence the jet stability and stretching during the electrospinning process. In addition to morphological changes, the presence of nanofillers induces a visible color shift, with GNP-containing mats acquiring a more grayish appearance, whereas CNT-filled systems tend toward a darker, nearly black coloration. Moreover, nanocomposite mats present a higher degree of morphological heterogeneity, arising both from the incorporation of carbon particles within the fibers and from the emergence of structural defects such as bead-like formations. CNT-filled systems generally promote the formation of thicker fibers, while GNP-containing mats exhibit the opposite trend, with reduced average fiber diameters, and hybrid-filled fibers showing intermediate behavior, as evidenced in Figure 5 and summarized in Table 2.

**Figure 5:**
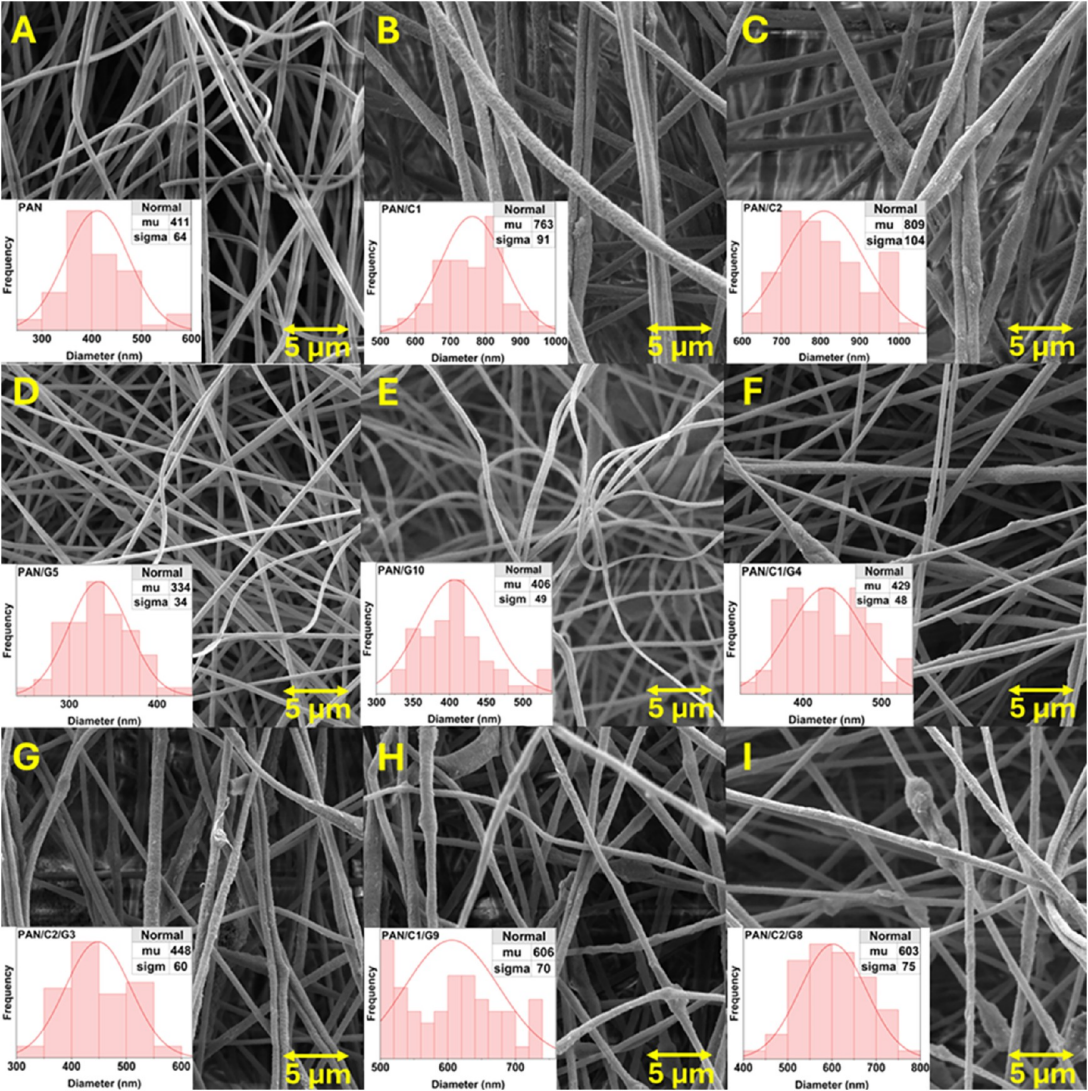
FEG-SEM images and average diameter distribution: (A)—PAN; (B)—PAN/C1; (C)—PAN/C2; (D)—PAN/G5; (E)—PAN/G10; (F)—PAN/C1/G4; (G)—PAN/C2/G3; (H)—PAN/C1/G9; (I)—PAN/C2/G8.

**Table 2:**
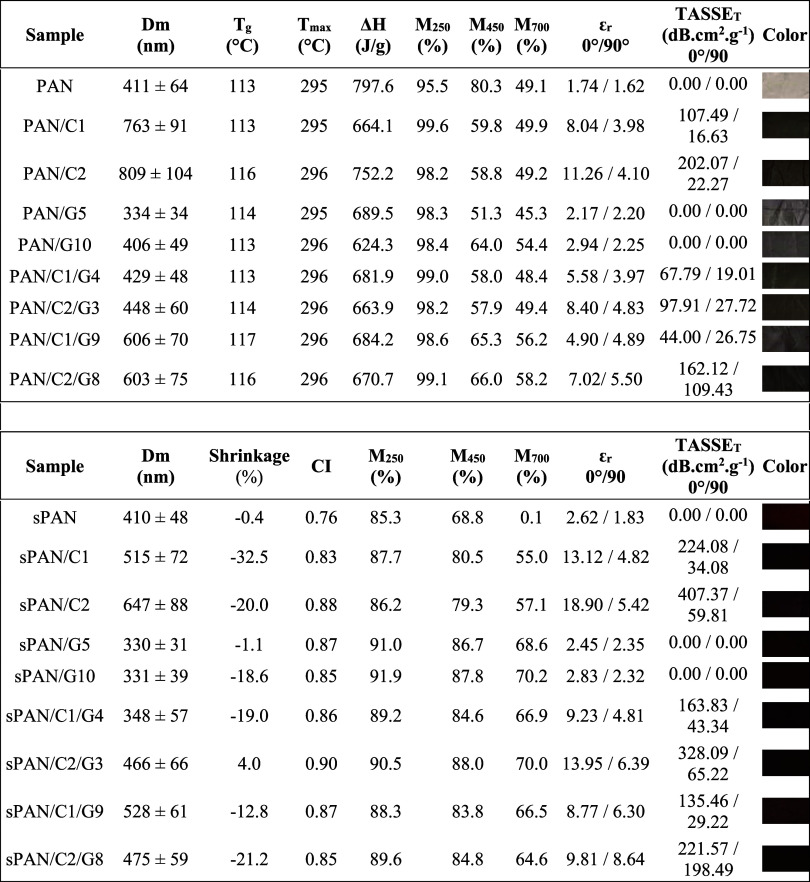
Summary of Main Properties Obtained for the Different Compositions as Electrospun^50^ and after Stabilization

After oxidative treatment, a reduction in fiber diameter is commonly observed, which is associated with cyclization, dehydrogenation, and oxidation reactions that remove free volume and induce contraction of the polymer backbone. Thermal exposure also promotes surface roughening due to volatile release and structural rearrangement. In addition, thermal shrinkage and stress gradients can cause some surface-exposed fibers to appear broken or partially severed. Interfiber bonding points are also observed, particularly at locations where fibers were initially in physical contact, indicating partial fusion during ladder structure formation. These morphological changes are accompanied by a distinct color transition: pristine PAN mats are white due to strong light scattering, whereas stabilized mats progressively change from yellow to brown and dark brown as conjugated aromatic structures develop.^47^ This gradual darkening confirms the successful formation of π-electron-rich ladder domains.

Morphological evidence from the FEG-SEM images (Figure 6A–I) demonstrates that both nanofiller type and concentration significantly influence the fiber geometry after the stabilization heat treatment. The neat PAN sample (Figure 6A, sPAN) exhibits smooth fibers with a mean diameter of 410 ± 48 nm (Table 2). In CNT-reinforced samples (Figure 6B,6C), protruding nanotubes and signs of fiber breakage are observed. Fiber rupture is attributed to insufficient annealing during stabilization, suggesting that slower heating rates may be required to allow greater fiber elongation and stress relaxation. In addition, PAN/CNT fibers display increased diameters, reaching 515 ± 72 nm for sPAN/C1 and 647 ± 88 nm for sPAN/C2, indicating that CNT incorporation alters jet stretching dynamics and final filament dimensions.

**Figure 6:**
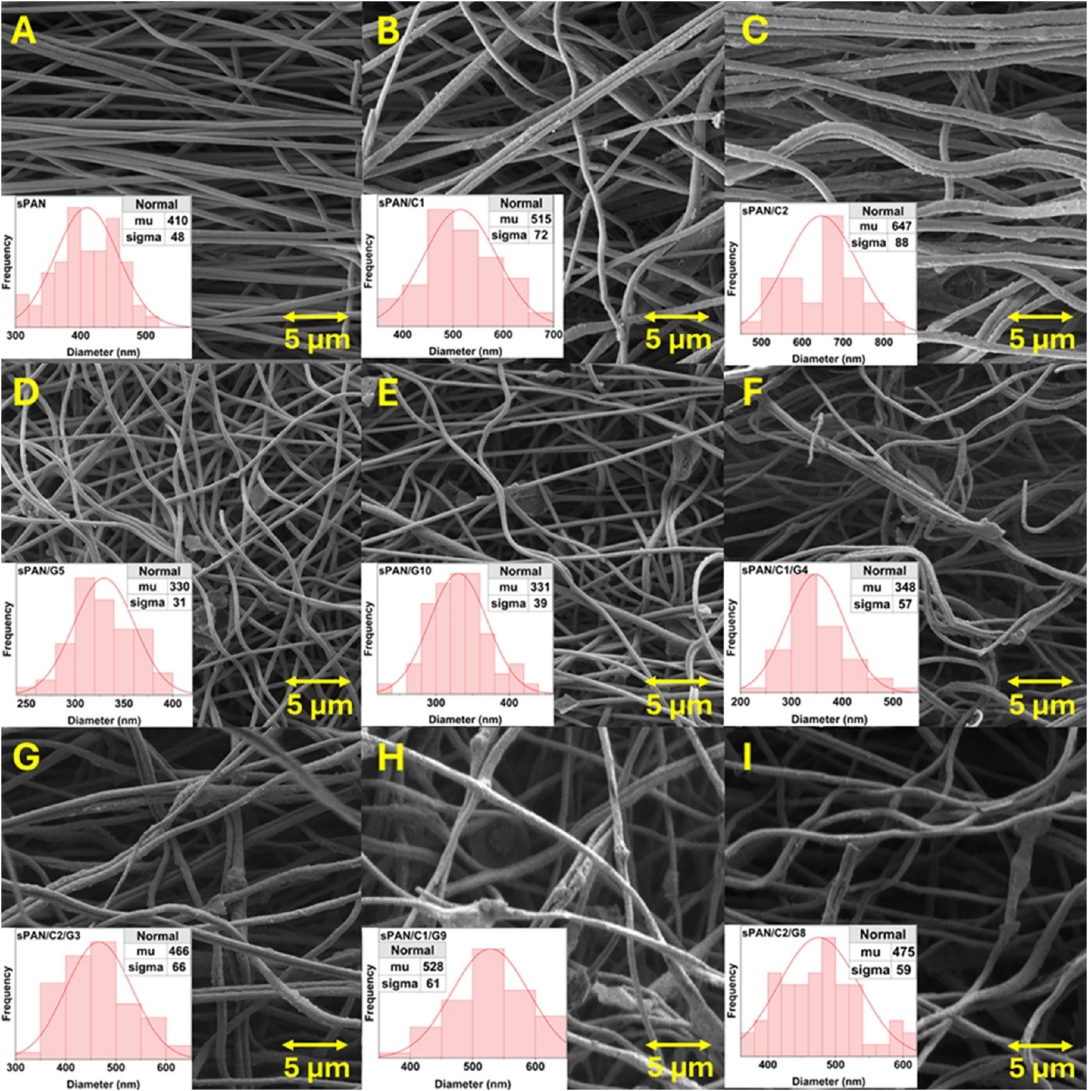
FEG-SEM images and average diameter distribution: (A)—sPAN; (B)—sPAN/C1; (C) sPAN/C2; (D)—sPAN/G5; (E)—sPAN/G10; (F)—sPAN/C1/G4; (G)—sPAN/C2/G3; (H)—sPAN/C1/G9; and (I)—sPAN/C2/G8.

In contrast, GNP-containing samples (Figure 6D,6E) exhibit thinner fibers, with average diameters of 330 ± 31 nm (sPAN/G5) and 331 ± 39 nm (sPAN/G10). This refinement suggests that GNPs increase solution conductivity and enhance jet elongation during electrospinning. Hybrid CNT/GNP systems display intermediate morphologies combining tubular and plate-like features within the same mat. Samples shown in Figure 6F,6I present fiber diameters between the single-filler trends: 348 ± 57 nm for sPAN/C1/G4, 466 ± 66 nm for sPAN/C2/G3, 528 ± 61 nm for sPAN/C1/G9, and 475 ± 59 nm for sPAN/C2/G8.

The relative variation in fiber diameter after stabilization further highlights how the nanofiller type influenced the thermo-oxidative shrinkage. Neat PAN exhibits negligible contraction (−0.4%). CNT-only mats undergo pronounced diameter reduction (−32.5% and −20.0%), indicating significant radial collapse and increased generation of internal stress, which may increase brittleness and favor pore and void formation. Shrinkage during PAN stabilization arises from the superposition of physical and chemical contributions. Physical shrinkage is associated with the entropic recovery of PAN chains that were frozen in highly oriented, low-entropy conformations during electrospinning. Upon heating below the onset of stabilization reactions, these chains relax toward thermodynamically favored, leading to dimensional contraction.^48^ Chemical shrinkage, which becomes dominant at elevated temperatures, is intrinsically linked to the cyclization of nitrile groups, resulting in chain stiffening, reduced molecular mobility, and an irreversible decrease in fiber length.^49^ While the initial cyclization-driven shrinkage is only weakly influenced by the surrounding atmosphere, the presence of oxygen promotes additional reactions at advanced stages of stabilization, thereby intensifying the overall chemical shrinkage. GNP-only mats show less pronounced shrinkage (−1.1% and −18.6%), consistent with more uniform chain rearrangement. Hybrid systems again exhibit intermediate behavior, with shrinkage values ranging from −21.2% to −12.8%. Notably, the sPAN/C2/G3 sample shows a positive diameter variation (+4.0%), suggesting a greater susceptibility to fiber fusion during stabilization.^26^

The orientation distribution was quantified from SEM micrographs and obtained using OrientationJ, as shown in Figure 7. Baseline level, peak center, integrated area, and fwhm, summarized in Table 3, were obtained through Gaussian fitting of the orientation distribution curves and are employed as quantitative descriptors of preferential alignment and angular dispersion.

**Figure 7:**
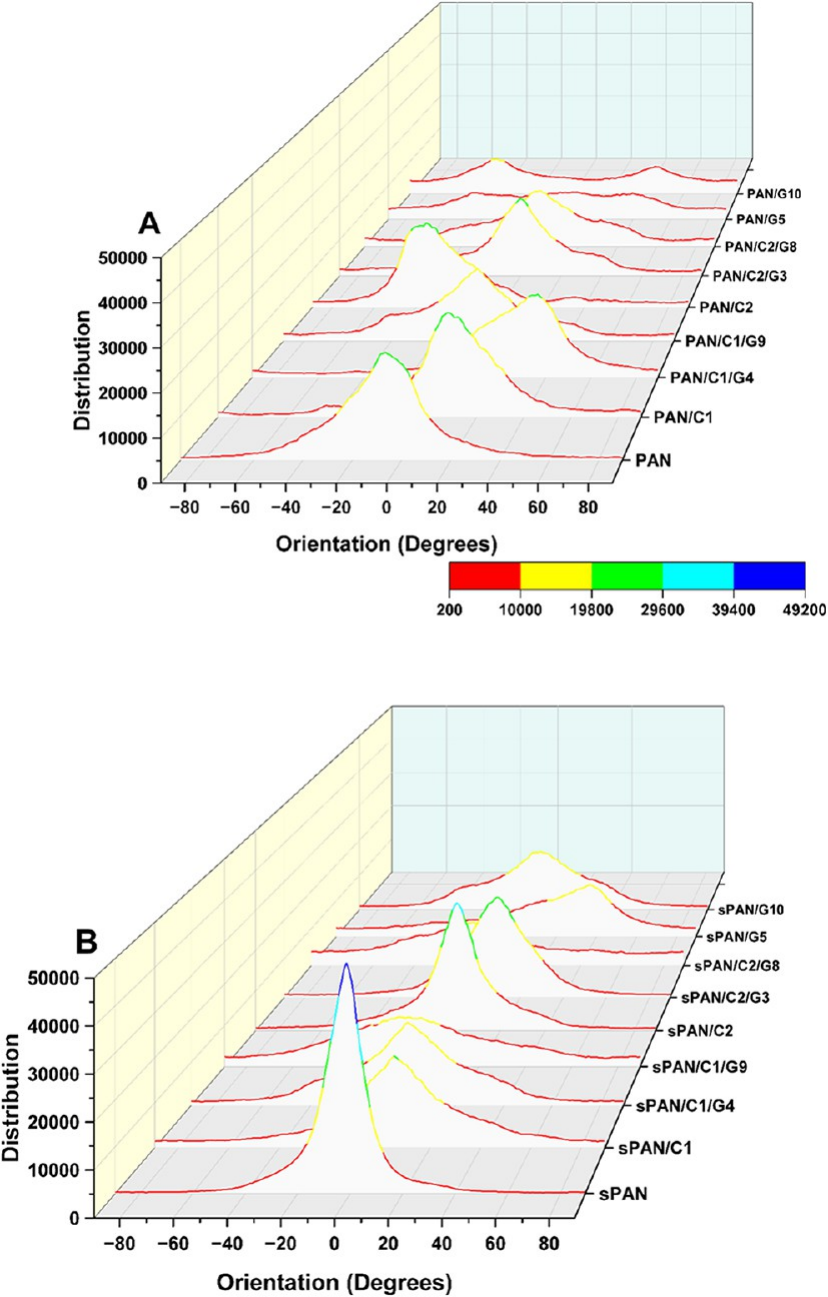
Orientation distribution of fiber orientation for: (A)—electrospun and (B)—stabilized mats.

**Table 3:** Summary of Main Parameters Obtained after Gaussian Fit for the Different Compositions as Electrospun and after Stabilization

sample	peak	baseline	peak center	area	FWHM	adj. *R*-Square
PAN	1	1320.35 ± 105.86	–7.20 ± 0.21	8.08 × 10^05^ ± 1.28 × 10^04^	36.11 ± 0.55	0.977
PAN/C1	1	1565.29 ± 93.24	11.88 ± 0.18	7.65 × 10^05^ ± 1.08 × 10^04^	33.28 ± 0.46	0.981
PAN/C1/G4	1	1432.98 ± 90.46	29.93 ± 0.21	7.91 × 10^05^ ± 1.18 × 10^04^	42.01 ± 0.58	0.981
PAN/C1/G9	1	2030.80 ± 123.77	–2.55 ± 0.38	6.82 × 10^05^ ± 1.73 × 10^04^	48.13 ± 1.09	0.951
PAN/C2	1	1761.79 ± 122.47	–32.38 ± 0.26	7.29 × 10^05^ ± 1.46 × 10^04^	35.15 ± 0.68	0.963
PAN/C2/G3	1	2374.09 ± 121.56	0.60 ± 0.31	6.20 × 10^05^ ± 1.48 × 10^04^	36.50 ± 0.84	0.950
PAN/C2/G8	1	2118.91 ± 124.44	1.16 ± 0.42	6.65 × 10^05^ ± 1.86 × 10^04^	54.72 ± 1.30	0.949
PAN/G5	1	3252.36 ± 25.59	–42.12 ± 0.38	1.20 × 10^05^ ± 5.80 × 10^03^	29.97 ± 0.72	0.993
	2		4.31 ± 0.42	2.67 × 10^05^ ± 1.19 × 10^04^	53.37 ± 2.57	
	3		42.86 ± 0.29	7.50 × 10^04^ ± 6.43 × 10^03^	23.68 ± 0.92	
PAN/G10	1	4358.37 ± 36.46	–41.94 ± 0.19	1.76 × 10^05^ ± 3.35 × 10^03^	28.78 ± 0.52	0.973
	2		44.19 ± 0.26	8.61 × 10^04^ ± 2.77 × 10^03^	22.19 ± 0.69	
sPAN	1	1270.05 ± 134.19	–2.14 ± 0.11	8.19 × 10^05^ ± 1.14 × 10^04^	17.81 ± 0.26	0.979
sPAN/C1	1	2159.33 ± 96.10	7.66 ± 0.25	6.58 × 10^05^ ± 1.22 × 10^04^	39.40 ± 0.69	0.970
sPAN/C1/G4	1	1310.82 ± 119.54	1.44 ± 0.32	8.11 × 10^05^ ± 1.73 × 10^04^	51.75 ± 0.96	0.967
sPAN/C1/G9	1	2390.75 ± 73.94	–9.58 ± 0.28	6.17 × 10^05^ ± 1.20 × 10^04^	64.70 ± 0.99	0.980
sPAN/C2	1	1911.81 ± 145.49	1.03 ± 0.22	7.00 × 10^05^ ± 1.48 × 10^04^	25.34 ± 0.55	0.956
sPAN/C2/G3	1	1135.88 ± 66.86	9.22 ± 0.12	8.42 × 10^05^ ± 7.98 × 10^03^	35.04 ± 0.32	0.992
sPAN/C2/G8	1	3716.22 ± 41.08	–16.05 ± 0.23	3.78 × 10^05^ ± 5.76 × 10^03^	48.43 ± 0.66	0.982
sPAN/G5	1	2977.16 ± 110.77	27.91 ± 0.46	5.09 × 10^05^ ± 1.55 × 10^04^	48.19 ± 1.31	0.930
sPAN/G10	1	1005.21 ± 109.65	3.36 ± 0.29	8.64 × 10^05^ ± 1.70 × 10^04^	58.89 ± 0.95	0.976

Pristine PAN exhibits a low baseline (≈1320), a peak centered at −7.20°, a high integrated area (8.08 × 10^5^), and a moderate width (FWHM of 36.11°), indicating a dominant orientation population aligned with the electrospinning direction and limited background randomness. The incorporation of CNTs modifies this distribution without disrupting its unimodal nature. At 1 wt % CNT (PAN/C1), the peak shifts to 11.88°, while the baseline increases slightly (≈1565) and FWHM showed a mild reduction (33.28), with the integrated area remaining on the same order of magnitude (7.65 × 10^5^). At 2 wt % CNT (C2), a more pronounced peak displacement occurs (−32.38°), evidencing reorientation of the preferential axis. Although the area remains high (7.29 × 10^5^), the baseline increases further (≈1762), indicating greater random contribution superimposed on the principal orientation. The fwhm (35.15°) remains comparable to pristine PAN, demonstrating that CNT addition primarily perturbs alignment direction but maintains a coherent, predominantly unimodal orientation profile.

In contrast, GNP-only systems exhibit fundamentally different behavior. Both G5 and G10 display markedly elevated baselines (≈3250–4358), reflecting a strong increase in randomly oriented fibers. Their distributions fragment into multiple orientation populations, with peak centers located around ±42°, and for PAN/G5, near 0°, and integrated areas substantially lower than PAN and PAN/CNT systems. The coexistence of multiple peaks and the reduced area associated with each confirm that the dominant single-orientation population observed in PAN is reduced. Thus, planar GNPs promote angular splitting and diminish global alignment coherence, generating a more heterogeneous and spatially dispersed orientation structure.

Hybrid CNT/GNP mats retain an overall Gaussian-adjustable profile but progressively inherit the more random character introduced by GNPs. While their integrated areas often remain comparable to PAN (10^5^–10^6^ order), baselines increase relative to CNT-only systems and FWHM values broaden substantially, frequently exceeding 40–50°. This behavior reflects the redistribution of fiber populations within a broader angular range. The slight reduction of the adjusted *R* ^2^ values (∼0.95 in most GNP concentrated hybrids) further indicates that a single Gaussian only approximates a more complex orientation distribution.

Thermal stabilization systematically modifies the orientation distribution, although the extent of this change depends on the composition. During this stage, the ladder structure of PAN undergoes nitrogen elimination and structural rearrangements that favor the formation of thermodynamically stable pentagonal and heptagonal rings in addition to the dominant hexagonal network. These structural irregularities generate curvature and distortions within the evolving carbon planes, contributing to a reduction in chain alignment.^33^ Such misalignment can be mitigated by the application of external stress, which restrains structural relaxation and suppresses the development of curvature.^51^ In systems without externally imposed global order, carbon-based fillers may act as anchoring sites that locally hold the polymer chains, thereby reducing the effects of entropic shrinkage. Nevertheless, the results indicate that PAN systems containing carbon particles exhibit a lower degree of alignment compared with the stabilized PAN sample. This behavior suggests that when molecular orientation is predominantly induced by mechanical drawing, the anchoring effect of carbon particles constrains the cooperative rearrangement of adjacent PAN chains. Consequently, rather than promoting additional ordering, the presence of these fillers may hinder further alignment and, at specific locations, locally oppose the stress induced orientation process.^17^

For instance, peak centers tend to displace toward a preferential axis, evidencing that the traction applied during heat treatment promotes reorientation. This effect is particularly pronounced in the sPAN/C2 composition, which peak moves from −32.38° to 1.03°. Directional displacement is also observed for GNP-containing and hybrid systems. Alignment enhancement after stabilization is demonstrated by the significant fwhm narrowing for sPAN sample, where the width decreases from 36.11° to 17.81°, indicating strong angular concentration. An equally relevant example is the sPAN/C2 system, in which fwhm is reduced from 35.15° to 25.34°, confirming substantial improvement in orientation coherence.

In GNP-only mats, stabilization also promotes homogenization through reduced distribution complexity. Stabilized GNP mats no longer present as evident orientation subpopulations, indicating convergence toward a more unimodal distribution. Hybrid systems reinforce this interpretation. After stabilization, their adjusted R^2^ values increase compared with the electrospun state, although fwhm remains relatively broad due to the intrinsic dispersion introduced by planar GNPs.

### 3.3 FT-IR

During oxidative stabilization, PAN nanofibers undergo extensive molecular rearrangement that can be monitored by FT-IR spectroscopy, as shown in Figure 8. In the electrospun precursor mats, the spectra are dominated by the sharp nitrile stretching band at approximately 2240 cm^–1^, originating from aligned −C≡N groups along the semicrystalline PAN backbone. Additional characteristic PAN absorptions are also observed, including CH_2_/CH stretching between the 2940–2860 cm^–1^ region and CH bending modes between 1450 and 1350 cm^–1^.^52^

**Figure 8:**
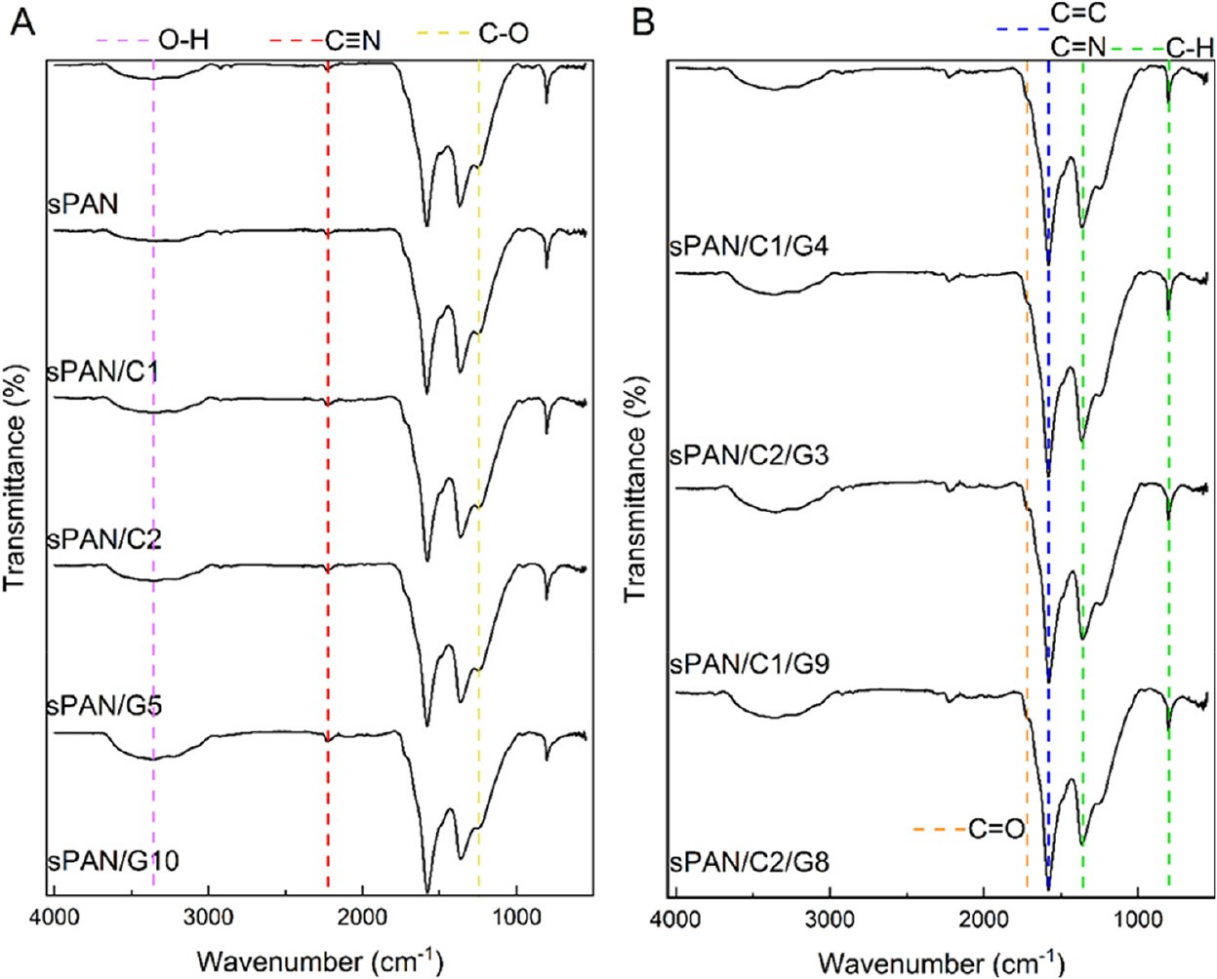
FT-IR spectra: (A) sPAN, sPAN/C1, sPAN/C2, sPAN/G5, and sPAN/G10, and (B) sPAN/C1/G4, sPAN/C2/G3, sPAN/C1/G9, and sPAN/C2/G8.

The onset and progression of cyclization are evidenced by the gradual attenuation and broadening of the nitrile band, resulting from the intramolecular conversion of nitrile groups into conjugated C=N and aromatic structures.^53^ This transformation is further supported by the market reduction of CH_2_ deformation band near 1450 cm^–1^ and the emergence of an intense absorption around 1580 cm^–1^, attributed to C=C and C=N stretching vibrations within the developing aromatic ring systems. Additional bands near 804 and 1374 cm^–1^, assigned to out-of-plane C–H deformation in aromatic environments and vibrations associated with hydrogen bonded to newly formed carbon–carbon double bonds, respectively, further confirm the advancement of stabilization reactions.^54^

Other spectral changes corroborate the progressive loss of aliphatic character. The weakening of absorptions at approximately 2933 and 2872 cm^–1^, corresponding to asymmetric and symmetric CH_2_ stretching modes, along with the decline of the C–C stretching band close to 1077 cm^–1^, reflects the breakdown of linear polymer backbone segments and the formation of a conjugated ladder structure.^55^ Together, these spectral features are consistent with extensive cyclization and dehydrogenation reactions accompanying the transition of PAN from a thermoplastic polymer into an infusible, thermally stable network.

Oxygen incorporation during stabilization introduces additional functional groups that further modify the infrared spectra.^56^ The appearance of a band near 1240 cm^–1^, is attributed to C–O stretching vibrations formed during oxidative cross-linking. At the same time, a broad signal around 3200 cm^–1^ indicates the formation of hydroxyl groups, while shoulder-like absorptions between 1730 and 1680 cm^–1^ suggest the presence of carbonyl functionalities. These oxygen-containing groups enhance intermolecular cohesion and thermal stability, facilitating subsequent carbonization by promoting CN oligomerization through nucleophilic pathways, accelerating nitrile conversion via radical propagation mechanisms, and facilitating the condensation of aromatic domains.

To compare the influence of the different stabilized mats on the chemical structure of the fibers, a quantitative analysis of the FT-IR spectra was performed. The conjugation index (CI) was calculated using eq 4, based on the relative intensities of the band at 1600 cm^–1^, assigned to C=C stretching in the thermo-oxidatively stabilized mats, and the band at 2240 cm^–1^, corresponding to C≡N stretching in the nonstabilized mats. The CI values obtained from the FTIR analysis are summarized in Table 2.

Residual nitrile groups observed in the stabilized neat PAN sample are attributed to the atactic stereochemical structure of the polymer.^57^ In such systems, unreacted nitrile groups are likely isolated within hydrogen-poor segments that are not favorably positioned for cyclization. Their complete removal generally requires pyrolytic treatment at higher temperatures. In contrast, the incorporation of carbon nanofillers markedly enhances stabilization efficiency. A plausible explanation for this behavior involves the presence of surface functionalities on the carbon particles, particularly oxygen-containing groups, which can locally promote oxidative reactions during stabilization through their thermal decomposition.^18^

In addition, carbon-based nanoparticles can enhance heat dissipation across the nanofiber mat during thermal exposure.^58^ When highly thermally conductive fillers are incorporated into a polymer matrix, their elevated structural order and crystallinity contribute to improved phonon transport within the composite. This reduces phonon scattering and increases the phonon mean free path, thereby promoting more efficient heat conduction.^59^

A substantial improvement in thermal conductivity was reported by Khan et al., who prepared PAN solutions containing CNTs and graphene nanoflakes for electrospinning. The incorporation of CNTs increased the thermal conductivity by more than 10-fold, reaching 0.24 W·m^–1^·K^–1^ at a filler loading of 16 wt %.^60^ In comparison, the addition of graphene nanoflakes enabled thermal conductivity values as high as 2.7 W·m^–1^·K^–1^ at a concentration of 8 wt %, highlighting the strong effectiveness of graphene in promoting heat transport within polymer fibers.^61^

Moreover, Behdinan et al. numerically evaluated the steady-state and transient heat transfer behavior of functionally graded polymer cylinders reinforced with graphene or CNTs.^58^ Their findings demonstrated that increasing nanofiller content significantly shortens the time required to reach thermal equilibrium and intensifies internal temperature gradients. Overall, graphene-reinforced systems exhibited superior thermal performance compared to CNT-reinforced composites, owing to their higher effective thermal conductivity.

Therefore, it is expected that the addition of carbon load could favor intramolecular cyclization over polymer chain scission and other degradation pathways, thereby increasing the overall extent of conjugation. Notably, hybrid nanofiller systems exhibit the highest CI values, indicating a synergistic effect between CNTs and GNPs. These results demonstrate that hybrid-reinforced PAN nanofibers achieve more effective thermo-oxidative stabilization than their single-filler or unfilled counterparts.

### 3.4 Thermal Behavior

PAN nanofiber mats exhibit a characteristic thermal signature under an inert atmosphere that reveals the fundamental chemical events involved in the precursor conversion. In the pristine state, DSC curves display a dominant exothermic peak between 250 and 300 °C, which is primarily associated with the initiation of intramolecular cyclization. At the molecular level, PAN chains composed of linear −CH_2_–CH­(CN)– repeating units undergo radical-mediated cyclization, forming conjugated C=N structures and initiating an autocatalytic reaction process.^40^ Because DSC measurements are conducted under a nitrogen atmosphere, the heat released during this transformation is not dissipated by oxidative reactions, resulting in a sharp and highly energetic exothermic peak. The cyclization temperature and enthalpy for all studied samples before stabilization are summarized in Table 2.

After oxidative stabilization, this characteristic DSC event is drastically attenuated or nearly eliminated, as shown in Figure 9A,9B. Stabilization consumes nitrile functionalities and induces cyclization, hydrogen abstraction, cross-linking, and aromatization, converting the linear PAN precursor into a rigid ladder-type polymer with delocalized π systems. Consequently, the stabilized architecture exhibits substantially enhanced thermal resistance, enabling safe progression toward subsequent carbonization without fiber melting or fusion.^62^

**Figure 9:**
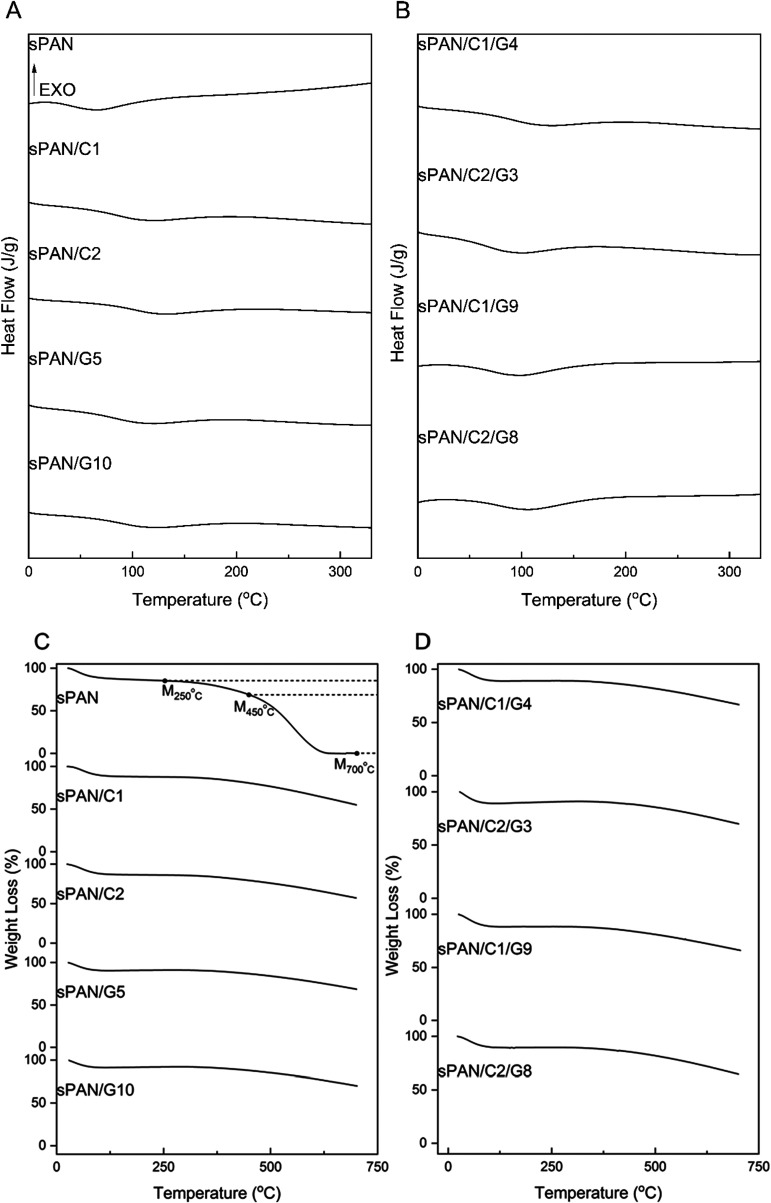
DSC and TGA curves, under N_2_ atmosphere: (DSC: A and C)—sPAN, sPAN/C1, sPAN/C2, sPAN/G5, and sPAN/G10; (TGA: B and D)—sPAN/C1/G4, sPAN/C2/G3, sPAN/C1/G9, and sPAN/C2/G8.

TGA further clarifies these structural transformations. In untreated PAN mats, weight loss occurs in multiple steps: below ∼250 °C, associated with the removal of moisture, residual solvent, and low-molecular-weight species. A major degradation event between 250 and 450 °C, driven by intense intramolecular reactions such as cyclization, dehydrogenation, chain scission, and volatile species (e.g., H_2_, H_2_O, HCN, NH_3_, CO, CO_2_, and light hydrocarbons).^63^ In the absence of a stabilizing network, pristine PAN partially melts and depolymerizes during this stage.

Following oxidative stabilization, this steep weight loss event between 250 and 450 °C is significantly reduced because most nitrile groups have already been converted into thermally robust ladder structures. Thermal degradation then proceeds more gradually, particularly above 500–600 °C, as nitrogen-, hydrogen-, and oxygen-containing volatiles are slowly released and turbostratic carbon domains begin to form.^64^ Importantly, the stabilized precursor maintains fibrous morphology, enabling carbon microstructure development along the original fiber axis

Figure 9C,9D illustrate these thermal differences, while Table 2 summarizes residual mass values at 250, 450, and 700 °C. Until 250 °C, all nonstabilized samples maintained very high mass retention (above 95%), reflecting a mostly intact linear polymer structure presenting unreacted nitrile groups and residual solvent. After stabilization, however, a systematic reduction in weight at 250 °C was observed for every composition before thermal treatment: neat sPAN decreased from 95.5% to 85.3%, sPAN/C1 from 99.6% to 87.7%, and sPAN/C2 from 98.2% to 86.2%, while GNP- and hybrid-reinforced samples showed smaller but still significant reductions (sPAN/G5 from 98.3% to 91.0%, sPAN/G10 from 98.4% to 91.9%, sPAN/C1/G4 from 99.0% to 89.2%, sPAN/C2/G3 from 98.2% to 90.5%, sPAN/C1/G9 from 98.6% to 88.3%, sPAN/C2/G8 from 99.1% to 89.6%). This initial weight loss is consistent with FT-IR results, which indicate that stabilization was not complete and that a fraction of nitrile groups remained uncyclized. Therefore, part of the polymer chains preserved a linear structure. In this temperature region, the release of these light species contributes to the observed mass reduction.

In the precursor state, PAN mats exhibit weight loss values ranging from 15.2% to 47.0%, indicating the onset of thermal degradation within the 250–450 °C temperature range. After oxidative stabilization, this behavior is altered, with the stabilized samples showing weight losses of up to only 16.6% over the same interval. This substantial reduction reflects the formation of a thermally robust molecular architecture, capable of withstanding elevated thermal stress, thereby confirming that the stabilization process converts PAN into an infusible and thermally stable structure.

At 700 °C, neat PAN exhibits the lowest char yield, retaining only 0.1% of the original weight of the sample. In other words, practically no carbonaceous residue was formed during the TGA analyses, indicating inefficient carbon formation. This behavior is due to the sample not being stabilized during the TGA analysis, because it was conducted under an inert atmosphere. The incorporation of CNTs increased char yield, reaching 55.0% and 57.1% for sPAN/C1 and sPAN/C2, respectively. Nonetheless, CNT dispersion remains challenging due to strong van der Waals-driven agglomeration, such clusters act as structural defects and can reduce effective carbon conversion when compared to GNP. Mats reinforced solely with GNPs showed improved residue (45.3% to 68.6% for sPAN/G5 and 54.4% to 70.2% for sPAN/G10), supporting their role in promoting structural reinforcement and the formation of graphitic domains. Hybrid systems exhibited comparable or superior performance: sPAN/C2/G3 and sPAN/C1/G9 mats retain 70.0% and 66.5% of mass at 700 °C, respectively. Meanwhile sPAN/C2/G8 sample showed mass retention of 64.6% at the same temperature. These results demonstrate the synergistic role of GNPs in improving CNT dispersion and creating more homogeneous conductive networks, ultimately enhancing char yield during high-temperature treatment.

### 3.5 Electromagnetic Properties

A clear evolution in the frequency-dependent dielectric behavior is observed when comparing electrospun PAN mats before and after oxidative stabilization. In its pristine state, the dielectric response of electrospun PAN fibers is dominated by the orientation polarization of nitrile side-group dipoles along a saturated, linear aliphatic backbone. At low to intermediate frequencies, these dipoles, along with intrinsic polar defects, can partially align with the alternating electric field. Under these conditions, the imaginary component of permittivity is mainly associated with internal friction arising from localized dipolar rotation, resulting in relatively low dielectric losses and low overall permittivity.^65–67^

Oxidative stabilization fundamentally alters this response by converting the linear PAN into a heterocyclic ladder structure through cyclization, dehydrogenation, and cross-linking reactions. This transformation generates extended π-conjugated domains and increases the concentration of oxygen-containing functional groups. Consequently, stabilized mats exhibit increased electrical permittivity (ε_r_), arising from enhanced dipolar polarization of newly formed functional groups and from interfacial polarization associated with the characteristic skin-core morphology of stabilized PAN fibers.^68^

This radial heterogeneity of PAN fibers develops progressively during thermal stabilization, as can be seen in Figure 10. Initially, precursor fibers are structurally uniform, showing no radial heterogeneity and only gradual densification as cyclization proceeds and the fiber diameter decreases.^69^ As stabilization progresses, the fiber surface experiences strong absorption of atmospheric oxygen (Figure 10A), which limits the growth of extended aromatic domains. Meanwhile, oxygen diffusing toward the interior promotes the transformation of cyclized structures in the core (Figure 10D) into more aromatic domains through consecutive dehydrogenation reactions, favored by higher temperatures in the fiber interior, while thermal energy is dissipated more easily at the skin.^38^ This results in the formation of a structurally distinct core. As the core expands, the chemical structure of the skin evolves, whereas the outermost surface remains comparatively unchanged. Because oxidation is diffusion-controlled, the formation of oxidized and cross-linked structures in the skin (Figure 10C) progressively restricts oxygen transport toward the interior.^25^

**Figure 10:**
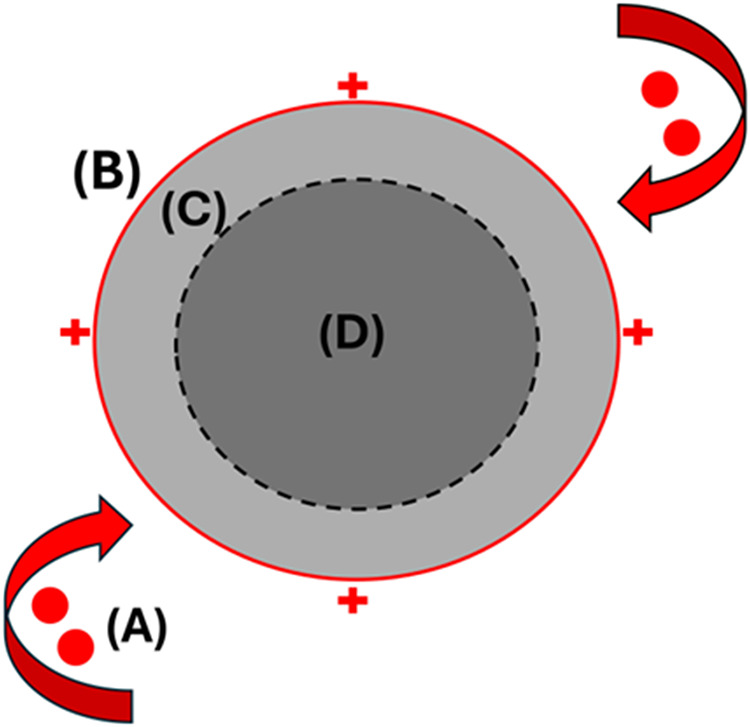
Schematic of skin–core structure of PAN stabilized fibers: (A)—oxygen absorption, (B)—fiber surface, (C)—skin, (D)—core.

Moreover, the delocalization of π-electrons within the aromatic rings contributes to electronic polarization and could provide conductive losses due to electron conduction by a hopping mechanism.^70^ These dielectric mechanisms are further intensified by the addition of CNTs and GNPs. These carbon nanomaterials can act as microcapacitor elements, increasing charge accumulation at interfaces and enhancing energy storage capability.^71^ Moreover, an increase in imaginary permittivity is expected due to ohmic losses in a conductive cluster formed by CNTs interconnected. Furthermore, the combined use of CNTs and GNPs can produce a complementary effect, whereby CNTs favor the formation of a conductive segment, while GNPs enhance interfacial polarization through their extended surface area.

Figure 11 and Table 2 summarize the dielectric permittivity values obtained for all samples in the X-band frequency range. The stabilized neat PAN mat exhibited relatively low permittivity, ranging from ε_r_ = 1.83 when the electric field is applied perpendicular to the fiber alignment (90°) to ε_r_ = 2.62 in the parallel configuration (0°), demonstrating intrinsic dielectric anisotropy similar to that observed for the untreated PAN mats. The incorporation of carbon nanofillers leads to a pronounced increase in permittivity, particularly when the electric field is aligned parallel to the fiber axis. For CNT-reinforced samples containing 1 and 2 wt % CNTs, ε_r_ reached 13.12 and 18.90 at 0°, compared to 4.82 and 5.42 at 90°, respectively, confirming preferential polarization and charge transport along the fiber direction.

**Figure 11:**
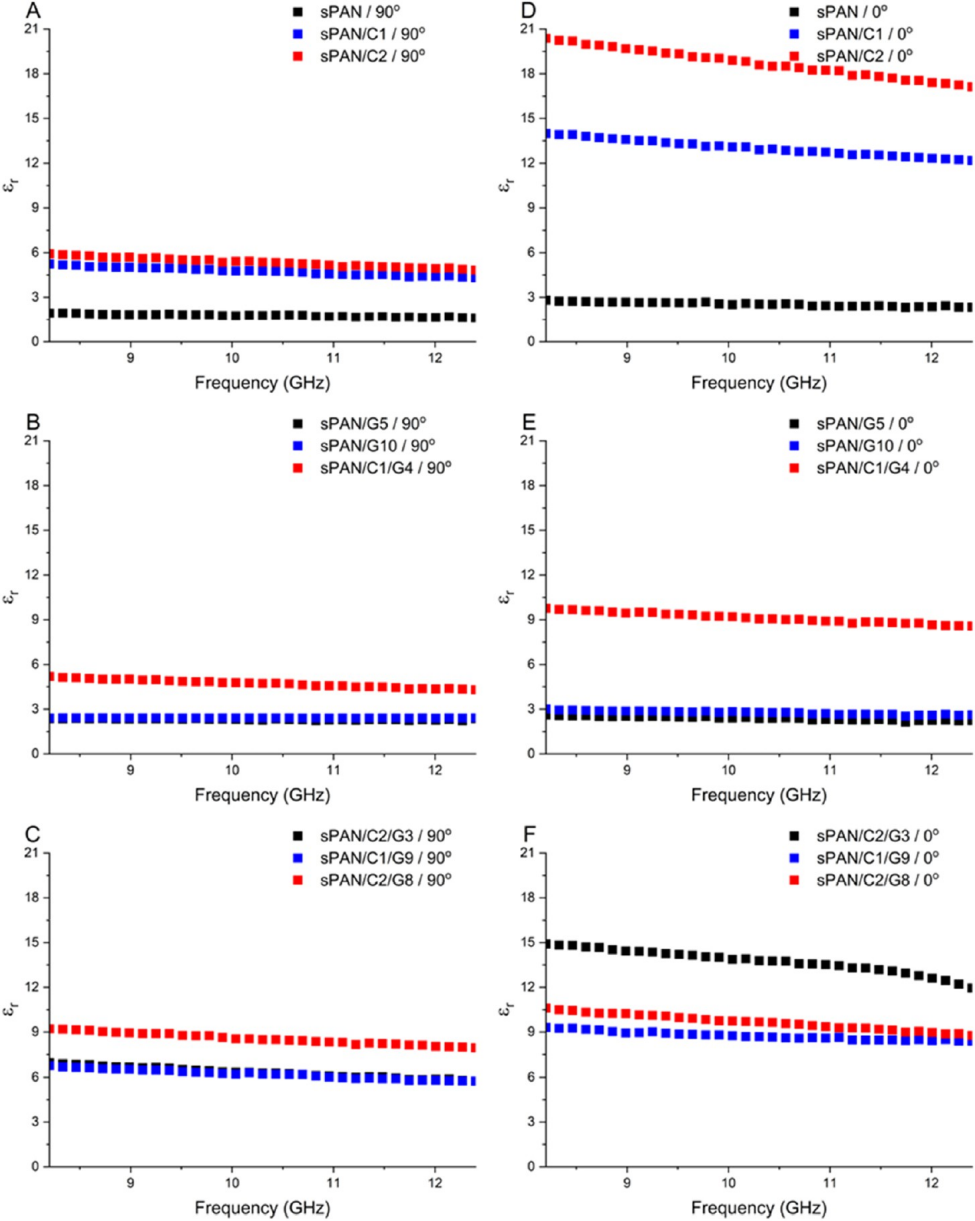
Resulting permittivity as a function of frequency for the compositions: (A, D)—sPAN, sPAN/C1 and sPAN/C2; (B, E)—sPAN/G5, sPAN/G10 and sPAN/C1/G4; (C, F)—sPAN/C2/G3, sPAN/C1/G9 and sPAN/C2/G8.

This pronounced directional dependence could arises during electrospinning, when the combined action of elongational forces and the applied electric field induces preferential axial alignment of both polymer chains and nanotubes, resulting in conductive segments predominantly oriented along the fiber direction. As the matrix itself remains nonconductive, efficient charge transport and polarization are favored along the fiber axis, whereas transverse directions remain comparatively insulating, resulting in the strong dielectric anisotropy observed in the CNT-containing stabilized mats.

In contrast, GNP-based mats display a much weaker dielectric response. The ε_r_ values range from 2.35 and 2.32 at 90°, and from 2.45 to 2.83 at 0° for mats containing 5 and 10 wt % GNPs, respectively, indicating that platelet fillers alone provide limited enhancement of polarization. This difference highlights the greater effectiveness of CNTs in increasing dielectric permittivity, owing to their extremely high aspect ratio and superior ability to form long-range conductive pathways.

Hybrid CNT/GNP mats exhibit intermediate behavior. Although ε_r_ increases significantly relative to neat PAN, the values remain lower than those obtained for systems composed only of CNT with the same total filler content. For instance, sPAN/C1/G4 and sPAN/C2/G3 mats exhibit ε_r_ values of 9.23 and 13.95 at 0°, compared to 4.81 and 6.39 at 90°, respectively, while sPAN/C1/G9 and sPAN/C2/G8 show ε_r_ values of 8.77 and 9.81 at 0°, and 6.30 and 8.64 at 90°.

As previously described, when a dielectric material is exposed to an electromagnetic field, its internal charge carriers undergo polarization. This process induces an internal electric field that opposes the applied EM wave, thereby reducing the transmission of the incident electromagnetic wave and contributing to reflection at the material interfaces.^42^ The intensity of this phenomenon is directly related to the magnitude and nature of the polarization mechanisms involved, including electronic, ionic, molecular dipolar, and interfacial polarization. In lossy materials, however, the response to the electromagnetic field extends beyond energy storage. Charge carriers move in response to the oscillating field and experience resistive and relaxation processes. These mechanisms hinder their motion and result in energy dissipation. Consequently, part of the electromagnetic energy is converted into thermal energy through dielectric and conductive losses, characterizing absorption.^72^ Both processes contribute to the overall attenuation of the electromagnetic wave and can be quantitatively described in terms of total shielding effectiveness SE_t_, absorption shielding effectiveness SE_a_, and reflection shielding effectiveness SE_r_, where SE_t_ represents the combined contribution of reflection and absorption mechanisms within the material.

For EMI materials that are aiming to be lightweight, the ratio of SE to density can be used to characterize their shielding performance, but the EMI SE can be easily increased by increasing the material thickness. Therefore, the thickness averaged specific SE (TASSE) can be used as an evaluation index of weight efficiency, which is defined as the EMI SE divided by area density (TASSE = SE/areal density).^43,73,74^ Under these conditions, TASSE_T_ of all samples are presented in Table 2 at 10 GHz, while reflection and absorption contributions are shown in Figure 12. PAN fibers and PAN/GNP composite fibers do not exhibit significant electromagnetic attenuation, regardless of orientation or thermal treatment. Consequently, these results are not included in the discussion.

**Figure 12:**
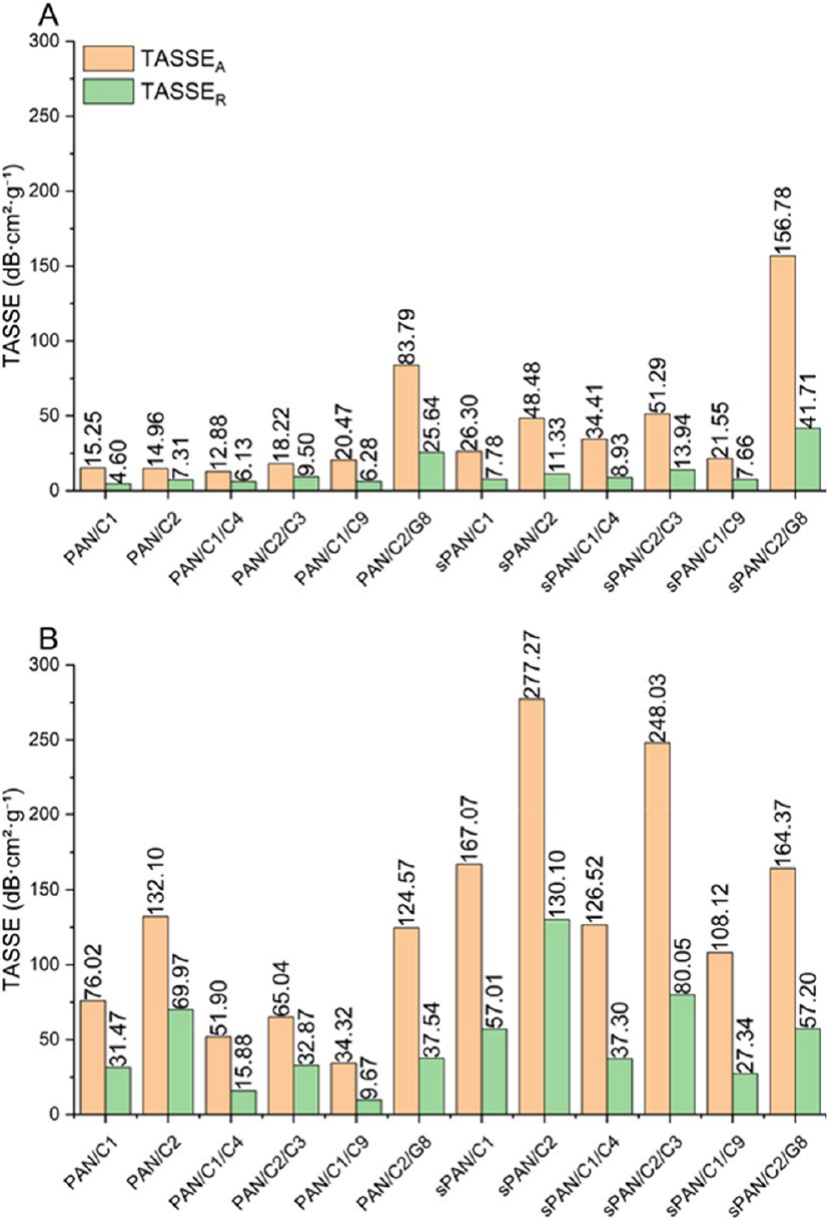
TASSE_R_ and TASSE_A_ at 10 GHz for samples with different orientations (A) 90° and (B) 0°.

In general, all samples exhibit predominantly absorptive behavior, regardless of the concentration and type of incorporated particles. This result indicates that the porous architecture of the electrospun mats contributes significantly to this performance. Due to the high porosity, a larger fraction of the incident electromagnetic energy penetrates into the interior of the material, which limits the reflected component.^75^ The high void fraction promotes multiple internal reflections, increasing the effective propagation path of the wave within the structure, intensifying dielectric loss mechanisms and, consequently, absorption.^76^

As anticipated from the permittivity results, the samples cut parallel to the collector rotation direction exhibit the lowest electromagnetic attenuation values. In this configuration, the performance of newly spun fibers improves with increasing filler content and the composite containing the highest combined concentration of CNTs and GNPs, PAN/C2/G8, shows the best results within this group (TASSE_A_ = 83.79 dB·cm^2^·g^–1^; TASSE_R_ = 25.64 dB·cm^2^·g^–1^). The remaining composites containing only CNTs and hybrids present very similar values to each other, indicating that, under this orientation, CNTs and GNPs show a synergistic behavior.

After thermal stabilization, the same overall trend is preserved but becomes more pronounced due to the enhancement of the dielectric properties induced by cyclization and the increased structural conjugation of PAN. Under these conditions, sPAN/C2/G8 exhibits the best performance among the samples of this group (TASSE_A_ = 156.78 dB·cm^2^·g^–1^; TASSE_R_ = 41.71 dB·cm^2^·g^–1^), demonstrating that the thermal treatment amplifies both absorptive and reflective contributions to electromagnetic shielding. This could be due to the better fiber contact and reduction of pore size, leading to higher density of charger carrier interacting with the electromagnetic field, leading to an increase in reflection contributions. Moreover, absorption could increase by resistive and dielectric mechanisms due to charge displacement.

The highest absolute shielding values, however, are obtained for the samples cut perpendicular to the collector rotation direction, where the field–structure interaction is more efficient. In contrast to the previous configuration, the best overall result is achieved by the fibers containing only CNTs, sPAN/C2, which reaches TASSE_A_ = 277.27 dB·cm^2^·g^–1^ and TASSE_R_ = 130.10 dB·cm^2^·g^–1^. This behavior indicates that when the structural orientation favors charge transport along conductive segments, the CNT-only system becomes more effective than the hybrid formulation. As it becomes evident that, for both CNT concentrations (1% and 2%), the incorporation of GNPs leads to a reduction in shielding performance for both as spun and stabilized configurations.

### 3.6 Impedance Spectroscopy

Impedance spectroscopy provides insight into the electrical response of PAN mats by measuring the complex impedance (*Z* = *R* + *jX*), which describes the opposition of a material to an alternating current and encompasses resistance effects (*R*) and frequency-dependent processes associated with energy storage, named capacitive reactance (*X*_C_) and inductive reactance (*X*_L_).^77,78^ Capacitive reactance is inversely proportional to frequency and originates from charge displacement, where energy is stored in an electric field. Inductive reactance, in contrast, arises from energy storage in a magnetic field generated by current flow and increases proportionally with frequency. The sign of the imaginary part of impedance provides a direct indication of which reactive mechanism dominates the electrical response, with *X*_C_ and *X*_L_ contributing to a negative and positive imaginary component, respectively.

The modules of *Z* as a function of frequency for all samples are shown in Figure 13. In electrically percolated composites, the formation of continuous conductive networks leads to a frequency-independent |*Z*| regime up to a critical frequency.^79^ Such behavior is not observed in electrospun samples, which show impedance values that decrease with frequency. This is due to electrical conduction being restricted to the solid fiber phase. Therefore, charge carriers must follow tortuous pathways around void regions, which further suppresses transversal conductivity. Moreover, effective electrical connectivity in composite mats containing CNTs and GNPs is limited by the fillers’ spatial distribution and orientation: CNTs tend to agglomerate at the fiber surface, while GNPs are largely embedded within the fiber bulk, both predominantly aligned along the fiber direction, as observed in FEG-SEM images. This morphology suppresses the formation of the long-range three-dimensional transport.

**Figure 13:**
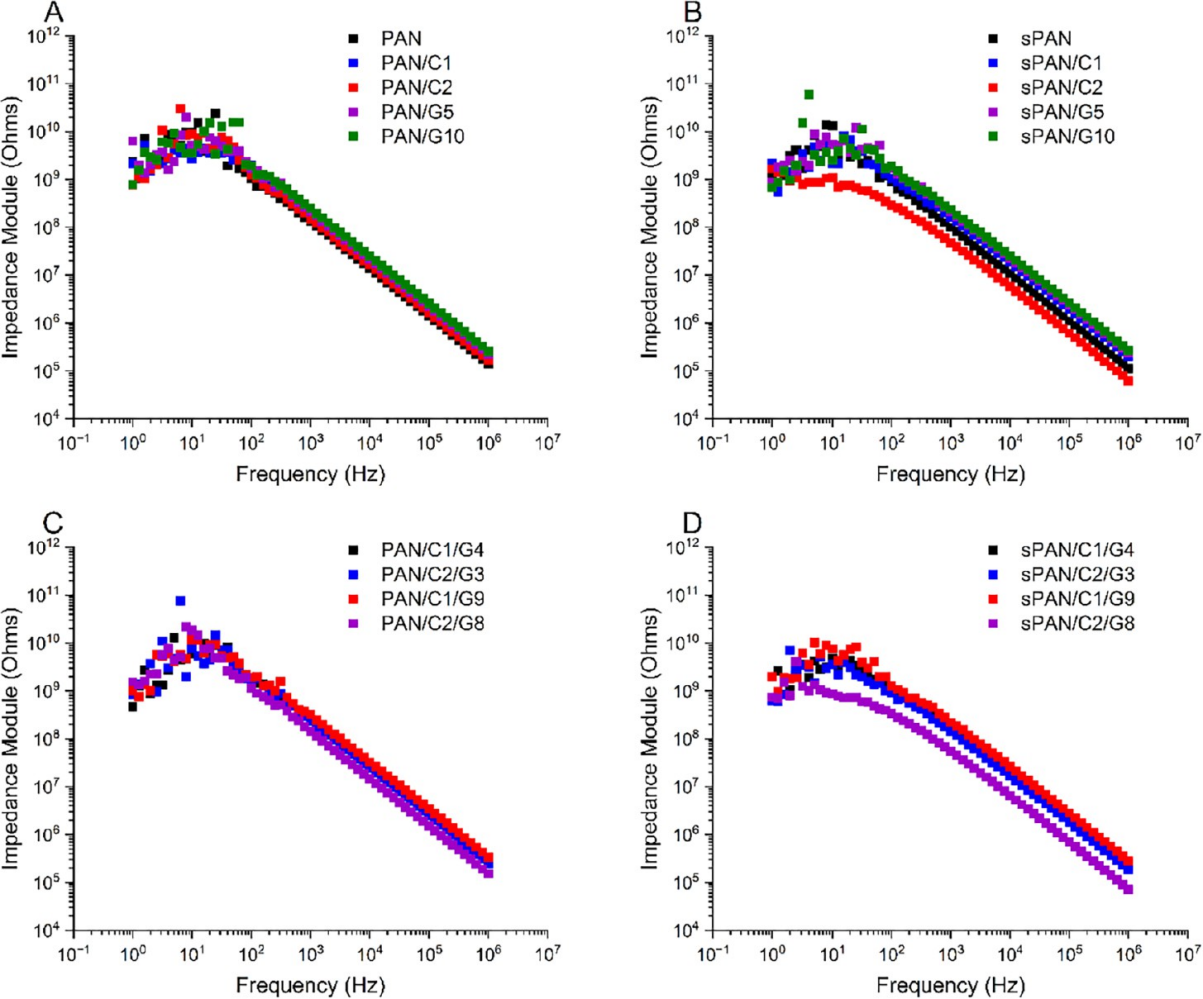
Impedance modulus as a function of frequency for all compositions.

As a result, PAN mats exhibit a predominantly capacitator-like response, due to shorter-range transport becoming increasingly dominant and capacitive reactance weakens, reflecting the progressive limitation of charge carriers’ responses as the time available for polarization decreases.^45^ Although, inductive-like effects have been reported for conductive carbon nanofiller segments in proximity, enabling the formation of local AC loops at high frequencies.^21^

While the overall impedance module remains relatively high, mainly due to resistive contacts between fibers, the presence of heteroatoms, and the porous architecture of the stabilized mats, CNT-rich samples, such as sPAN/C2 and sPAN/C2/G8, exhibit initial impedance values approximately 1 order of magnitude lower, along with reduced dispersion and a more gradual decrease as frequency increases. This behavior could indicate that this formulation with high CNT loading (2 wt % CNTs) may be at the start of the electrical percolation threshold. At this critical concentration, conductive nanofillers that are initially dispersed as isolated domains within the insulating polymer matrix begin to form a continuous pathway spanning the material, enabling long-range charge transport.^80^

It is important to emphasize that the same concentration in the newly spun mats does not exhibit comparable behavior. This difference suggests that thermal stabilization, although not sufficient by itself to significantly increase intrinsic conductivity, can strongly influence the development of conductive segments. During stabilization, enhanced polymer chain mobility and structural rearrangement favor CNT reorganization, improving interparticle proximity and contact probability.^52^ This tendency is not observed for high GNP compositions (5 and 10 wt %), indicating the high aspect ratio of CNTs could present a greater statistical probability of network development at lower loading than plate-like geometry like GNPs, typically requiring higher contents to reach a comparable result.

## 4 Conclusions

This study shows that oxidative stabilization affected not only the chemical conversion of PAN but also the structural organization of electrospun nanofiber mats in an orientation-dependent manner. Fiber diameter decreased after stabilization due to densification and ladder structure formation. Alignment analysis showed that externally applied tension enhanced preferential orientation in some systems, whereas carbon nanoparticle incorporation partially reduced this effect by restricting chain mobility and limiting structural rearrangement. Successful cyclization and oxidative reactions were evidenced by the disappearance of the cyclization exothermic peak in DSC. Nanofiller incorporation also increased the conjugation index, while GNP-containing systems exhibited the highest char retention, confirming their effectiveness in promoting ladder structure consolidation and improving char yield. Stabilization also altered the electrical response. After thermal treatment, the impedance decreased by approximately 1 order of magnitude for sPAN/C2 and sPAN/C2/G8, indicating improved charge transport and suggesting that CNTs can establish conductive pathways within the fibrous network. From a functional perspective, TASSE depended on the interaction between filler distribution and structural anisotropy. Stabilization enhanced shielding performance by increasing dielectric response and intensifying polarization and conductive loss mechanisms. The highest TASSE values were obtained for sPAN/C2 under parallel field alignment, whereas hybrid systems showed superior performance under perpendicular orientation compared with single filler materials. Overall, these results indicate that electromagnetic shielding in electrospun PAN systems results from the combined effects of stabilization-induced structural evolution, filler dispersion, and macroscopic orientation.
